# Characterization and Regulation of the Amino Acid Transporter SNAT2 in the Small Intestine of Piglets

**DOI:** 10.1371/journal.pone.0128207

**Published:** 2015-06-24

**Authors:** Guangran Li, Jianjun Li, Bie Tan, Jing Wang, Xiangfeng Kong, Guiping Guan, Fengna Li, Yulong Yin

**Affiliations:** 1 Observation and Experiment Station of Animal Nutrition and Feed Science in South-Central China, Ministry of Agriculture, Hunan Provincial Engineering Research Center for Healthy Livestock and Poultry Production, Key Laboratory of Agro-ecological Processes in Subtropical Region, Institute of Subtropical Agriculture, Chinese Academy of Sciences, Changsha, Hunan 410125, China; 2 College of Life Sciences, Hunan Normal University, Changsha 41008, China; 3 University of the Chinese academy of sciences, Beijing 10008, China; 4 Hunan Collaborative Innovation Center for Utilization of Botanical Functional Ingredients, Changsha, Hunan 410000, China; 5 College of Bioscience and Biotechnology, Hunan Agricultural University, Changsha 410128, China; Humboldt-University Berlin, GERMANY

## Abstract

The sodium-dependent neutral amino acid transporter 2 (SNAT2), which has dual transport/receptor functions, is well documented in eukaryotes and some mammalian systems, but has not yet been verified in piglets. The objective of this study was to investigate the characteristics and regulation of SNAT2 in the small intestine of piglets. The 1,521-bp porcine full cDNA sequence of SNAT2 (KC769999) from the small intestine of piglets was cloned. The open reading frame of cDNA encodes 506 deduced amino acid residues with a calculated molecular mass of 56.08 kDa and an isoelectric point (*p*I) of 7.16. Sequence alignment and phylogenetic analysis revealed that SNAT2 is highly evolutionarily conserved in mammals. SNAT2 mRNA can be detected in the duodenum, jejunum and ileum by real-time quantitative PCR. During the suckling period from days 1 to 21, the duodenum had the highest abundance of SNAT2 mRNA among the three segments of the small intestine. There was a significant decrease in the expression of SNAT2 mRNA in the duodenal and jejunal mucosa and in the expression of SNAT2 protein in the jejunal and ileal mucosa on day 1 after weaning (*P* < 0.05). Studies with enterocytes in vitro showed that amino acid starvation and supplementation with glutamate, arginine or leucine enhanced, while supplementation with glutamine reduced, SNAT2 mRNA expression (*P* < 0.05). These results regarding the characteristics and regulation of SNAT2 should help to provide some information to further clarify its roles in the absorption of amino acids and signal transduction in the porcine small intestine.

## Introduction

In young piglets, the process of weaning may contribute to morphological and functional changes in the small intestine [1]. Early weaning stress has been shown to be associated with marked alterations in both amino acid metabolism [2] and genes related to macronutrient metabolism and cell proliferation in the gut [3].

Dietary amino acids are a major fuel for the small intestinal mucosa and are necessary for maintaining the intestinal mucosal morphology and function in neonatal piglets [4]. It has been demonstrated that amino acid availability regulates cellular physiology by modulating gene expression and signal transduction pathways [5]. A growing body of evidence suggests that some amino acid transporters may not only transport amino acids, but may also participate in nutrient signaling by sensing extracellular amino acids [6]. Sodium-dependent neutral amino acid transporter 2 (SNAT2, encoded by the gene *SLC38A2* (solute carrier family 38, member 2)) exhibits a dual transporter/receptor (“transceptor”) function and has been demonstrated in lower eukaryotes and some mammalian cells [7–9]. Sequences of *SLC38A2* have been detected in human, rat and mouse [10–12], but have yet to be cloned in pigs. Furthermore, little is known about the characteristics or regulation of SNAT2 in the small intestine of piglets. Therefore, the aim of the present study was to clone the full sequence of *SLC38A2* and explore the distribution and ontogenetic expression of SNAT2 during the suckling and post-weaning periods, as well as the effects of substrates on the expression of SNAT2 in the small intestine, which should help to further elucidate the role of SNAT2 in the absorption of amino acids and signal transduction in the porcine small intestine.

## Materials and Methods

### Animals and Experimental Design

All animals used in this study were treated humanely according to the Chinese Guidelines for Animal Welfare. The experimental protocol was approved by the Animal Care and Use Committee of the Chinese Academy of Sciences[9].

In this experiment, 64 neonatal piglets (Duroc × (Landrace × Large Yorkshire)) from 8 litters (8 piglets per litter) were assigned to 8 groups on the basis of different litter origins and similar body weights[10]. All piglets were housed in an environmentally controlled farrowing cage with hard plastic slatted flooring and had free access to drinking water[11]. Some piglets were nursed by sows until they were 21 days old, while other piglets were weaned until they were 14 days old and then housed in the same farrowing cage without a sow and fed creep feed (Artificial milk 101, Anyou Feed, China). Eight piglets from each litter were slaughtered on days 1 (s-day1), 7 (s-day7), 14 (s-day14) and 21 (s-day21) of age during the suckling period and on days 1 (w-day15), 3 (w-day17), 5 (w-day19) and 7 (w-day21) post-weaning. After electrical stunning, piglets were killed and the small intestine was rinsed thoroughly with ice-cold physiological saline solution. A segment of the duodenum (2 cm) was cut and fixed in 4% formaldehyde for immunohistochemical analysis and samples of the duodenal, jejunal and ileal mucosa were immediately snap-frozen in liquid nitrogen and stored at -80°C for RNA extraction and western blot analysis[12].

### Cell Culture and Treatment

Dulbecco’s modified Eagle’s F12 Ham medium (DMEM/F12) and Earls’ Balanced Salt Solution (EBSS, containing 123 mM NaCl, 26 mM NaHCO_3_, 5 mM KCl, 1.8 mM CaCl_2_, 1 mM NaH_2_PO_4_, 0.8 mM MgSO_4_, 5 mM Glucose, pH 7.4) were purchased from Hyclone (GE Healthcare Life Sciences, Logan, UT). Fetal bovine serum (FBS) and penicillin/streptomycin were purchased from Gibco (Grand Island, NY). Insulin-Transferrin-Selenium (ITS) was obtained from ScienCell (Carlsbad, CA), and plastic culture plates were manufactured by Corning Inc. (Corning, NY). Unless otherwise indicated, all other chemicals were purchased from Sigma-Aldrich (St. Louis, MO). Intestinal porcine epithelial cells (IPEC-1) were a generous gift from Dr. Guoyao Wu (Department of Animal Science, Texas A&M University).

IPEC-1 were routinely maintained in plastic culture flasks (100 mm^2^) at 37°C under 5% CO_2_ and cultured in DMEM/F12 supplemented with 5% FBS, 1% ITS, 5 μg/L epidermal growth factor (EGF), and 1% penicillin/streptomycin (P/S). The medium was changed every 2 days[13]. To study the effects of individual amino acids on the abundance of SNAT2 mRNA, cells were trypsinized and seeded in 6-well cell culture plates. At 90% confluence, cells were incubated for 8 h in EBSS for an amino acid-free (AA-) condition or in EBSS supplemented with amino acid mix (AA+) or individual amino acids (0.25 mM Glycine, 0.05 mM L-Alanine, 0.699 mM L-Arginine hydrochloride, 0.05 mM L-Asparagine-H_2_O, 0.05 mM L-Aspartic acid, 0.1 mM L-Cystine 2HCl, 0.05 mM L-Glutamic Acid, 2.5 mM L-Glutamine, 0.15 mM L-Histidine hydrochloride-H_2_O, 0.451 mM L-Leucine, 0.499 mM L-Lysine hydrochloride, 0.116 mM L-Methionine, 0.15 mM L-Proline, 0.25 mM L-Serine, 0.449 mM L-Threonine or 0.0442 mM L-Tryptophan) based on the formulation of DMEM/F12 for cell stimulation as described previously in neocortical neurons [14] and L6 cells [8]. Cells were sampled using the Trizol reagent (Invitrogen, Carlsbad, CA) for mRNA extraction [15].

### Molecular Cloning of SNAT2 from Piglets

For the amplification of specific homologs encoding SNAT2 from piglets, two degenerate primers were designed based on the reported conserved regions of SNAT2 in a database [16–17]. Total RNA was isolated from jejunal mucosa of newborn piglets using the Trizol reagent (Invitrogen, Carlsbad, USA) and the first-strand cDNA was synthesized from 1 μg of total RNA using the PrimeScript II 1st Strand cDNA Synthesis Kit (Takara Biotechnology (Dalian) Co., Ltd, Dalian, China) according to the manufacturer's protocol. The cDNA was then used as a template for PCR amplification using the *TaKaRa Ex Taq*. The PCR conditions were as follows: initial denaturation at 98°C for 10 min, 30 cycles of 95°C denaturation for 30 s, 58°C annealing for 30 s, 72°C extension for 30 s, and final extension ended at 72°C for 10 min. The resulting PCR fragments were subcloned, sequenced and compared with the reported SNAT2 sequences in the NCBI database. In all cases, PCR amplification products were gel-purified with an Agarose Gel DNA Purification Kit (Takara Biotechnology (Dalian) Co., Ltd, Dalian, China), ligated into the pMD 19-T Vector (Takara Biotechnology (Dalian) Co., Ltd, Dalian, China) and then transformed to competent E.coli DH5α (Takara Biotechnology (Dalian) Co., Ltd, Dalian, China). Plasmids harboring target fragments were isolated and were sequenced from both strands. All the primers used are shown in the Table 1.

**Table 1:** Primers used for PCR or qPCR.

Gene	Primers	Application
SLC38A2-F1	CTACTAGCTGCACCCGATTCCT	PCR
SLC38A2-R1	GAGACTCGTGGTTTTGTTGTTCATAT	PCR
SLC38A2-F2	ACGTTGCTGTGGTCTGTAACC	qPCR
SLC38A2-R2	CATTAAGATCGCAGGCACGATA	qPCR
β-actin-F1	GGATGCAGAAGGAGATCACG	qPCR
β-actin-R1	ATCTGCTGGAAGGTGGACAG	qPCR

### Real-time Quantitative PCR

The expression of SNAT2 mRNA was determined by real-time quantitative PCR. Total RNA was extracted from the mucosa or cell samples with the Trizol reagent (Invitrogen, Carlsbad, CA), treated with gDNA Eraser and 5×g DNA Eraser Buffer (Takara Biotechnology (Dalian) Co., Ltd, Dalian, China) according to the manufacturer’s instructions, and quantified by electrophoresis on 1% agarose gel and measuring optical density at 260 and 280 nm. First strand cDNA was synthesized with 5 × PrimeScript Buffer 2 and PrimeScript reverse transcriptase Enzyme Mix 1 (Takara Biotechnology (Dalian) Co., Ltd, Dalian, China). Primers were designed with Primer 5.0 (PREMIER Biosoft International, Palo Alto, CA) according to the gene sequence of pig to produce an amplification product (Table 1). Beta-actin was used as a housekeeping gene to normalize target gene transcript levels[18–19]. The resulting cDNA was diluted and used as a PCR template to evaluate gene expression. The reaction was performed in a volume of 10 μL (ABI Prism 7700 Sequence Detection System; Applied Biosystems, Foster City, CA). Briefly, 1 μL cDNA template was added to a total volume of 10 μL containing 5 μL SYBR Green mix, 0.2 μL Rox, 3 μL dH_2_O, and 0.4 mol/L each of the forward and reverse primers. The following protocol was used: (1) pre-denaturation program (10 s at 95°C); (2) amplification and quantification program, repeated 40 cycles (5 s at 95°C, 20 s at 60°C); (3) melting curve program (60–99°C with a heating rate of 0.1°C S-1 and fluorescence measurement). Cycle threshold (Ct) values are means of triplicate measurements. The comparative Ct value method was used to quantify expression levels of target genes relative to those for β-Actin[20–21]. Data are expressed relative to the values in piglets at d 1 or 14 or amino acid-supplemented (AA+) cells.

### Immunohistochemical Analysis

The expression of SNAT2 protein in the duodenal mucosa of piglets was determined using immunohistochemical analysis[22–23]. First, tissue pieces were fixed with 4% paraformaldehyde, and then serial paraffin-embedded sections were made. After being dewaxed in xylene and re-hydrated via a graded series of ethyl alcohol, the sections were heated by microwave in 0.01 mol/L citral acid solution for antigen retrieval and blocked with 4.5% hydrogen peroxide in phosphate-saline buffer for 15 minutes. The sections were incubated with the anti SNAT2 monoclonal antibody (1:300; Santa Cruz Biotechnology, Inc., Dallas, TX) overnight at 4°C and then washed 3 × 5 min in PBS and incubated with a SV Mouse or Rabbit Hypersensitivity Two-step Immunohistochemical Kit (Boster Biological Technology, Wuhan, China) overnight at 4°C according to the manufacturer’s instructions[24–25]. The sections were washed 3 × 3 min with PBS, followed by the addition of diaminobenzidine (Boster Biological Technology, Wuhan, China) as a chromogen for 3 to 5 min, which was strictly controlled under a microscope. Before staining, the primary antibodies were replaced by PBS as a negative control. After being rinsed under cold tap water for 5 min and counterstained with hematoxylin (Boster Biological Technology, Wuhan, China), sections were dehydrated through an alcohol gradient and covered by general clarity gum. The stained sections were reviewed and scored independently by 2 investigators using a microscope (Olympus, Tokyo, Japan). The expression of SNAT2 protein was expressed as the average optical density (the ratio of integral optical density to the area of tissue) in at least 5 areas that were randomly selected for counting at less than 200-fold magnification.

### Western Blot Analysis

The jejunal, ileal and duodenal mucosa samples were homogenized using RIPA Lysis Buffer (Beyotime Institute of Biotechnology, Shanghai, China) containing 50 mM Tris (pH 7.4), 150 mM NaCl, 1% Triton X-100, 1% sodium deoxycholate, 0.1% SDS, sodium orthovanadate, sodium fluoride, EDTA, leupeptin and 0.1 mM phenylmethylsulfonyl fluoride (PMSF) [26–28]. Protein concentrations were measured using the bicinchoninic acid assay method with BSA as a standard (Beyotime Institute of Biotechnology, Shanghai, China). All samples were adjusted to an equal concentration (50 μg protein). The supernatant fluid (containing tissue proteins) was then diluted with 4 × LDS Sample Buffer and 10 × Reducing Agent (Life Technologies Co., Carlsbad, CA) and heated at 70°C for 10 min. After the solution was cooled on ice, it was used for Western blot analysis. Briefly, aliquots of samples were loaded onto Bis-Tris Mini gels (Life Technologies Co., Carlsbad, CA). After separation on 4 to 12% gels, proteins were transferred to a polyvinylidene difluoride (PVDF) membrane (Millipore, Bedford, MA) under 200 mA for 2 h using an apparatus (Bio-Rad Transblot; Bio-Rad, Hercules, CA). The membranes were blocked in 5% fat-free dry milk in Tris-Tween buffered saline (TTBS; 20 mM Tris/150 mM NaCl, pH 7.5 and 0.1% Tween-20; Applygen, Beijing, China) for 2 h, and then incubated with these primary antibodies overnight at 4°C with gentle shaking: (SNAT2; 1:1000; Santa Cruz Biotechnology, Dallas, TX) or β-actin (1:1000; Cell Signaling Technology, Beverly, MA). After being washed 4 times with TTBS, the membranes were incubated at room temperature (25°C) for 2 h with secondary antibodies at 1:4,000 (horseradish peroxidase-conjugated goat anti-rabbit IgG; Boster Biological Technology, Wuhan, China). Finally, the membranes were washed 4 times with TTBS and then developed using a substrate (Supersignal West Dura Extended Duration Substrate; Pierce, Rockford, IL). The images were detected by chemiluminescence (Millipore, Billerica, MA). Each Western blot was subjected to multiple exposures to ensure that the chemiluminescence signals were linear. Western blots were quantified by measuring the intensity of correctly sized bands using Alpha Imager 2200 Software (Alpha Innotech Corporation, San Leandro, CA). All protein measurements were normalized to β-actin.

### Bioinformatics Analyses

Computer analysis of the DNA and amino acid sequences was carried out using the Basic Local Alignment Search Tool (BLAST) program, available from NCBI (http://blast.ncbi.nlm.nih.gov/Blast.cgi) [29]. Multiple alignments of the amino acid sequences of SNAT2 were performed using the Clustal W program in BioEdit software (Isis Pharmaceuticals, Inc., Carlsbad, CA).The isoelectric point and molecular mass of this protein were predicted from expasy (http://web.expasy.org/compute_pi/). The full-length SNAT2 protein sequence was phylogenetically analyzed using MEGA 6 software with a bootstrapping set of 1000 replicates [30]. The transmembrane region of porcine SNAT2 was predicted by TMHMM server 2.0 (http://www.cbs.dtu.dk/services/TMHMM/).

### Statistical Analysis

All statistical analyses were performed by one-way ANOVA using SPSS software 19.0 (SPSS Inc., Chicago, IL). The differences among treatments were evaluated using Ducan’s multiple comparison test. Probability values < 0.05 were taken to indicate statistical significance. All data are expressed as mean ± standard error of the mean (SEM).

## Results and Discussion

### Identification and Characterization of Porcine SNAT2 cDNA

**Fig 1:**
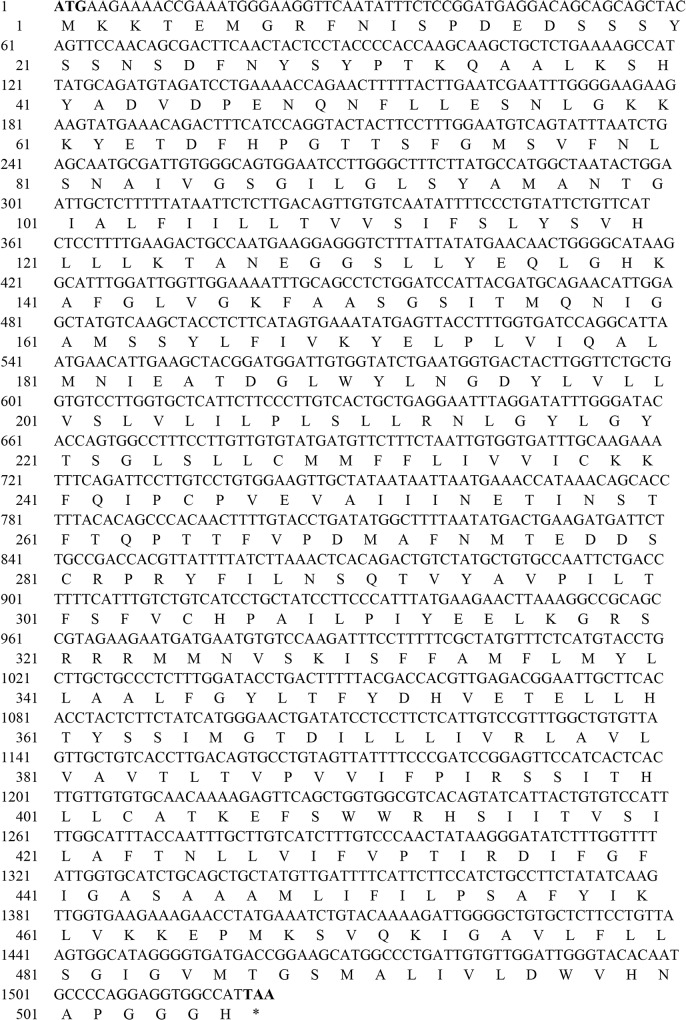
The nucleotide and deduced amino acid sequences of SNAT2 from Duroc × (Landrace × Large Yorkshire) piglets. CDS (coding sequence) is shown in capital letters and the deduced amino acid sequence is shown by single letter code of amino acids below the CDS. The start and termination codons are highlighted in bold. Two highly conserved domains: Aa_trans superfamily (residue range from 69 to 465) SdaC (residue range from 64 to 486).

The open reading frame (ORF) of *SLC38A2* from the small intestine of Duroc × (Landrace × Large Yorkshire) piglets was cloned and the sequence was added to GenBank (accession no. KC769999). The ORF of *SLC38A2* cDNA of 1,521 bp encoded a protein of 506 residues (Fig 1) with a calculated molecular mass of 56.08 kDa and an isoelectric point (*p*I) of 7.16. The alignment analysis showed that porcine *SLC38A2* mRNA sequence had 92%, 84% and 84% homology with known human (NM_018976.4), rat (NM_181090.2) and mouse (NM_175121.3) mRNA sequences, respectively. The porcine SNAT2 protein also shared 91%, 89% and 90% sequence identity with known human (NP_061849.2), rat (NP_851604.1) and mouse (NP_780330.2) protein sequences, respectively. These results indicate that porcine SNAT2 is highly evolutionarily conserved in mammals.

**Fig 2:**
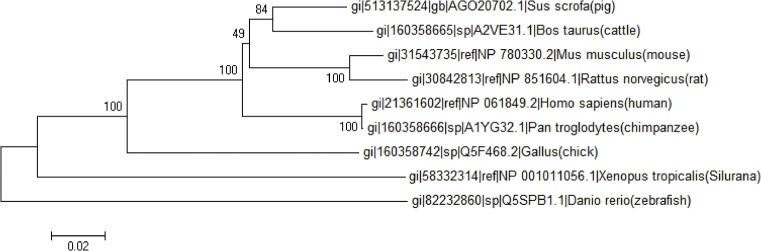
Phylogenetic tree of amino acid sequences of porcine SNAT2. Multiple sequence alignment was performed using Clustal W and the phylogenetic tree was constructed via the Neighbor-Joining method in MEGA 6 software. Bootstrap values from 1000 replicates for each branch were shown. NCBI accession numbers for each analyzed SNAT2 were indicated in parentheses. The scale bar is 0.02.

Conservation was also demonstrated by the phylogenetic analysis of protein (Fig 2). A phylogenetic tree of animal SNAT2 was constructed based on the multiple amino acid sequences, evolutionary analyses were conducted in MEGA6, and the evolutionary history was inferred using the Neighbor-Joining method [31]. The results revealed that porcine SNAT2 was closer to Bos Taurus SNAT2 and grouped into the same clade with 84 bootstrap support values (Fig 2). SNAT2 is highly evolutionarily conserved in animals, suggesting that the function of the domain assembly within SNAT2 is conserved.

**Fig 3:**
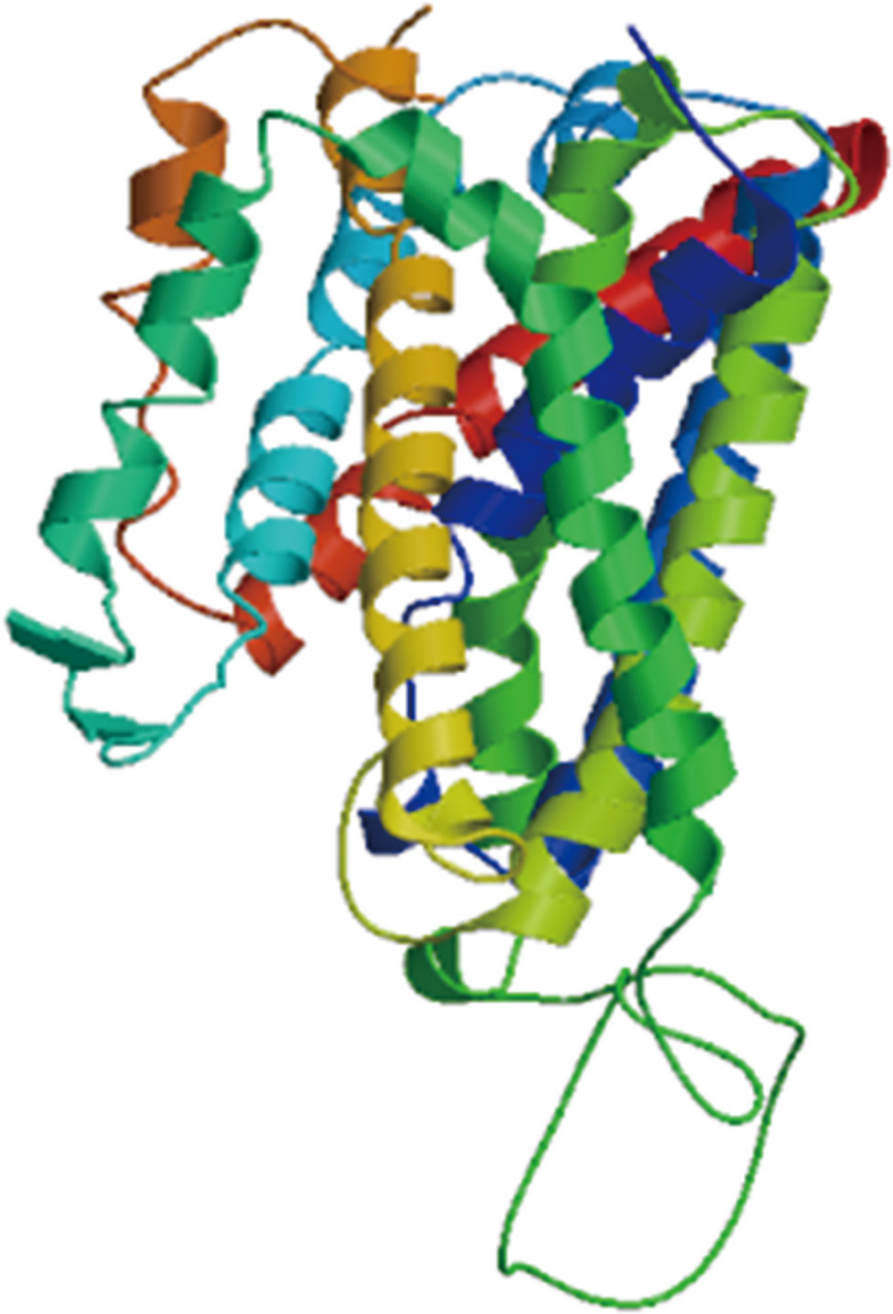
Three-dimensional structure of porcine SNAT2. The modeled residue range from 69 to 465. Based on template: 3gi8.1A. Sequence identity: 13.26%. The inaccuracy of per residue was visualized using a color gradient from blue (more reliable regions) to red (potentially unreliable regions).

As predicted by TMHMM2.0, the 506 deduced amino acid residues of SNAT2 contained 9 transmembrane regions. The protein contains two highly conserved domains: superfamily region: SLC5-6-like_sbd Superfamily (accession number: cl00456) and multi-domains: SdaC (accession number: *COG0814*). The results regarding the predicted three-dimensional structure of porcine SNAT2 are shown in Fig 3. The model was predicted based on a template: 3gi8.1A and the modeled residue range was from 69 to 465. The inaccuracy per residue was visualized using a 7-colour gradient from blue (more reliable regions) to red (potentially unreliable regions). The predicted 3D structure showed that the sequence identity between porcine SNAT2 protein and the template extracted from the SWISS-MODEL template library was only 13.26%. Some regions of the protein were disordered, unstructured or had flexible regions without a permanent regular secondary structure [32]. Fortunately, it has been suggested that disordered regions may possess biological functions, and could be involved in signaling and regulation processes [33–34]. However, further research is needed to fully understand the *SLC38A2* gene because the 5′ and 3′ untranslated regions are still unknown.

### Distribution and Ontogenetic Expression of SNAT2 mRNA in the Small Intestine of Suckling Piglets

SNAT2 is expressed ubiquitously in mammalian tissues and displays Na^+^-, voltage- and pH-dependent activity [35]. SNAT2 mRNA can be detected in the duodenum, jejunum and ileum of piglets by real-time quantitative PCR, which most likely reflects the reported system A activity [36]. During the suckling period from day 1 to 21, the duodenal mucosa had the highest SNAT2 mRNA abundance, which is similar to the trend in NHE-2 mRNA abundance in pigs [37]. The SNAT2 mRNA abundance in the jejunal mucosa on days 1 and 7 was higher, while on day 14 was lower, than that in the ileal mucosa during the suckling period of piglets (*P* < 0.05) (Table 2). The difference in the distributions of transporters in different segments of the intestine may be due to the unique morphological characteristics of the intestine, substrates and pH sensitivity [38]. Regarding the ontogenetic expression of SNAT2, it increased progressively from day 1 to 14 and then decreased on day 21 in the duodenum; the abundance in the jejunum on day 14 and that in the ileum on day 7 were the highest among those of days 1, 7, 14 and 21 (*P* < 0.05) (Table 2). This was in agreement with the changes in the expression of other reported amino acid and peptide transporters (e.g. NHE-2, b^0,+^AT and PepT1), which increased gradually from day 1 to the middle of the suckling period, and then gradually decreased from the middle to the end of the suckling period [17, 39]. Although amino acid profiles were not determined in the present study, this may reflect changes in absorbed nutrients and their metabolism with changes in the composition of colostrum or milk as well as changes in intestinal morphology and function [40]. The relationship between amino acid profiles and amino acid transporter expressions remains to be elucidated.

**Table 2:** *SLC38A2* mRNA abundance in small intestinal distribution of the suckling piglets from day 1 to 21^1^.

	duodenum	jejunum	ileum
Day 1	1.00±0.07^Ab^	0.45±0.07^Bb^	0.91±0.13^Ab^
Day 7	2.40±0.20^Aa^	0.62±0.09^Bb^	2.39±0.28^Aa^
Day 14	3.13±0.49^Aa^	1.91±0.36^Ba^	0.70±0.06^Cb^
Day 21	0.79±0.02^Ab^	0.42±0.13^Bb^	0.56±0.08^ABb^

^1^ The small intestinal mucosa samples were obtained from suckling piglets at days 1, 7, 14 and 21 of age. The expression of β-Actin was selected as an internal control in each real-time quantitative PCR. Data are expressed as the relative values to those of duodenum mucosa of 1 day old piglets. Values are mean ± SEM, n = 8. Values with different letters within the same row (^A-B^) or column (^a-b^) are different (*P* < 0.05).

### Changes in the Abundance of SNAT2 mRNA and Protein in the Small Intestinal Mucosa of Piglets within the First Week Post-weaning

**Fig 4:**
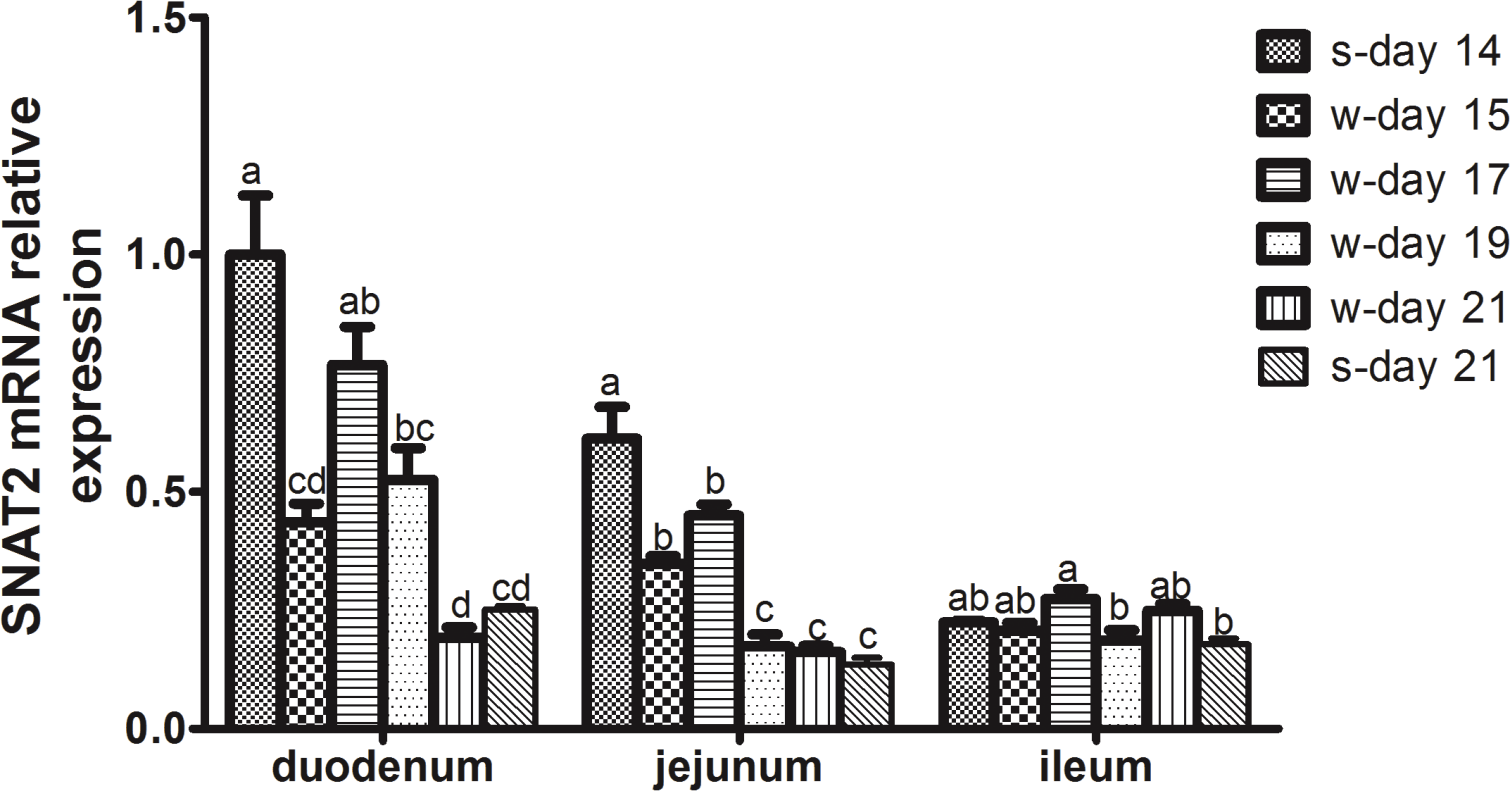
The relative SNAT2 mRNA expression in the small intestinal mucosa of piglets. The small intestinal mucosa samples were obtained from piglets at days 14 (s-day 14) and 21 (s-day 21) of age during suckling period and on day 1 (w-day 15), 3 (w-day 17), 5 (w-day 19) and 7 (w-day 21) post-weaning at 14 day of age. The relative SNAT2 mRNA expression was determined by real-time quantitative PCR using β-Actin as a reference gene. Data are expressed as the relative values to those of duodenum in suckling piglets on day 14. Values are means ± SEM, n = 8. ^a-d^ Values with different letters are significantly different (*P* < 0.05).

The changes in the abundance of SNAT2 mRNA and protein in the small intestinal mucosa of piglets on days 0 (s-day 14), 1 (w-day15), 3 (w-day17), 5 (w-day19) and 7 (w-day21) post-weaning were investigated. These values at day 21 (s-day 21) of age were also analyzed to examine the differential expression between suckling and weanling piglets of the same age. Within the first week post-weaning after 14 days of age, a sharp decline in the abundance of SNAT2 mRNA in the duodenal and jejunal mucosa was observed on the first day after weaning (day 15) (*P* < 0.05) (Fig 4). Although it is not known whether this change was due to age or weaning, weaning should play an important part in the decline in SNAT2 expression because there was a rebound increase on day 17 and then a decrease with increasing age (*P* < 0.05) (Fig 4). Disruption of the structural and functional mucosa associated with weaning should affect the absorption and utilization of nutrients [1], including amino acids, that are mainly mediated by specific transporters. It has been shown that early weaning resulted in the decreased expression of genes related to macronutrient metabolism in the gut [3], and dietary supplementation with glutamine, N-carbamylglutamate or glutamate could increase the expression of amino acid transporters in the intestine [4, 41, 42]. Gut mucosal amino acid transporter expression would ultimately affect the amino acid levels in circulation and their availability in visceral organs and peripheral tissues. Conversely, the transporters could adapt to the weaning process, which leads to the lack of a difference in the abundance of SNAT2 mRNA in all segments of the small intestinal mucosa between suckling and weanling piglets on day 21 (Fig 4). To clarify the effects of weaning on the expression of SNAT2, further studies will be needed to investigate the differential expression between the suckling and weanling piglets at days 15, 17, 19 and 21 of age.

**Fig 5:**
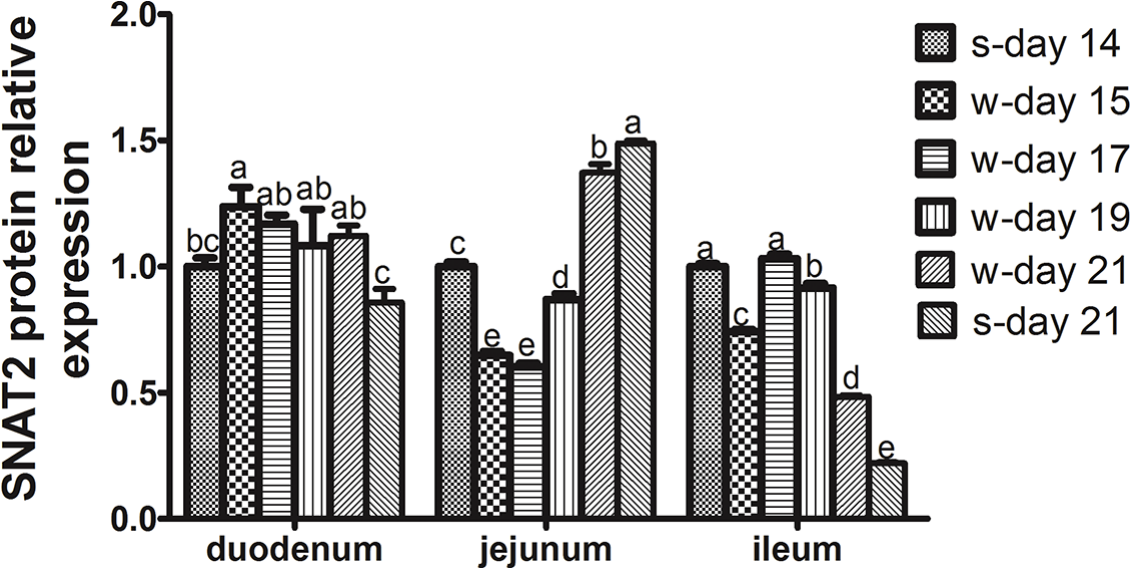
The relative SNAT2 protein expression in the small intestinal mucosa of piglets. The small intestinal tissue pieces or mucosa samples were obtained from piglets on days 14 (s-day 14) and 21 (s-day 21) of age during suckling period and on days 1 (w-day 15), 3 (w-day 17), 5 (w-day 19) and 7 (w-day 21) post-weaning at 14 day of age. The relative SNAT2 protein expression was determined by immunohistochemical or western blot analysis. Data are expressed as the relative values to those of duodenum in suckling piglets on day 14. Values are means ± SEM, n = 8. ^a-e^ Values with different letters are significantly different (*P* < 0.05).

**Fig 6:**
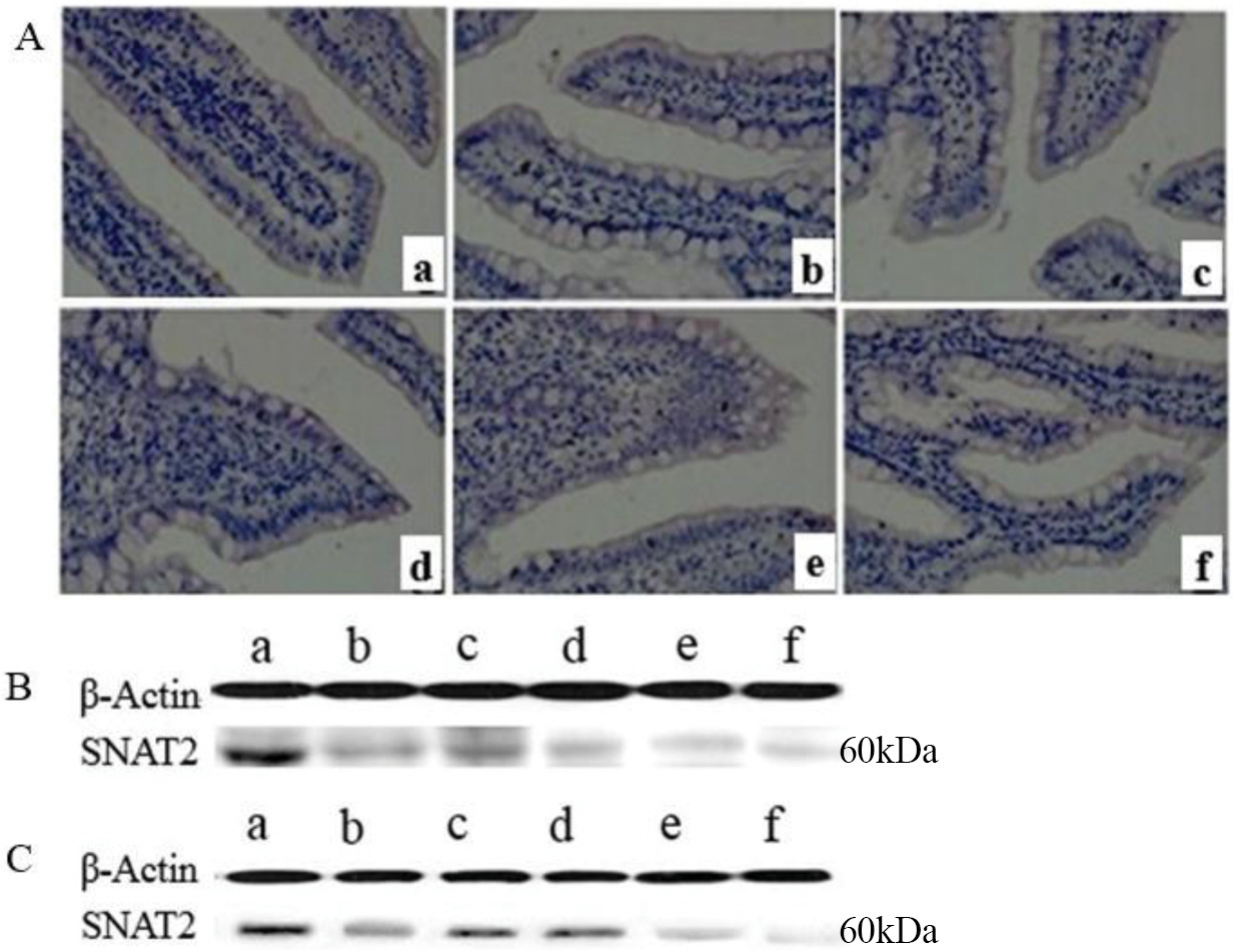
The representative images of immunohistochemical staining and western blot. (A) Immunohistochemical staining (magnification ×200) in duodenum, (B) Western blot in jejunum and (C) Western blot in ileum of piglets at days 14 (a) and 21 (f) of age during suckling period and on days 1 (b), 3 (c), 5 (d) and 7 (e) post-weaning at 14 day of age.

The changes in SNAT2 protein expression were not the same as those in gene expression in the small intestinal mucosa of piglets, as plotted in Fig 5. Representative results of immunohistochemical staining and western blotting for the protein are illustrated in Fig 6. In duodenum, the expression of SNAT2 protein was increased on day 1 after weaning compared with day 14 of age and the expression in weanling piglets was higher than that in suckling piglets at day 21 of age (*P* < 0.05). In the jejunum, there were significant decreases in the relative protein expression of SNAT2 on days 1, 3 and 5 after weaning compared with that at day 14 of age and a higher expression in suckling piglets than in weanling piglets at day 21 of age (*P* < 0.05). In the ileum, the protein expression of SNAT2 on days 1 and 7 after weaning was the lowest within 1 week post-weaning, and the expression in suckling piglets was lower than that in weanling piglets at day 21 of age (*P* < 0.05). These different patterns of mRNA and protein expression for different amino acid transporters have also been observed in several previous studies [43–45]. Fan et al. [43] demonstrated that changes in the maximal uptake activity and the abundance of EAAC1 protein and EAAC1 mRNA level did not follow a consistent pattern in proliferating and differentiating porcine neonatal small intestinal epithelia. Yang [45] showed that postnatal decreases in the jejunal apical abundances of B^0^AT1 and ASCT2 were likely regulated at the post-transcriptional level during the early suckling period, but the continued decline in the abundance of B^0^AT1 and ASCT2 could largely occur at both the transcriptional and translational levels through the weaning transition and the growing phase. Nevertheless, SNAT2 mRNA and protein expression generally showed downward trends in response to weaning, but not at the same age (Figs 4 and 6), which likely reflects decreases in the intake of nutrients. In view of the hybrid transceptor functions of SNAT2, to better understand the mechanisms of amino acid utilization by the intestines of piglets, we must further investigate the role of SNAT2 in the utilization of amino acids for gut mucosal growth via the mTOR signaling pathway [8].

### Regulatory Effects of Amino Acid Substrates on the Abundance of SNAT2 mRNA in Enterocytes of Piglets

**Fig 7:**
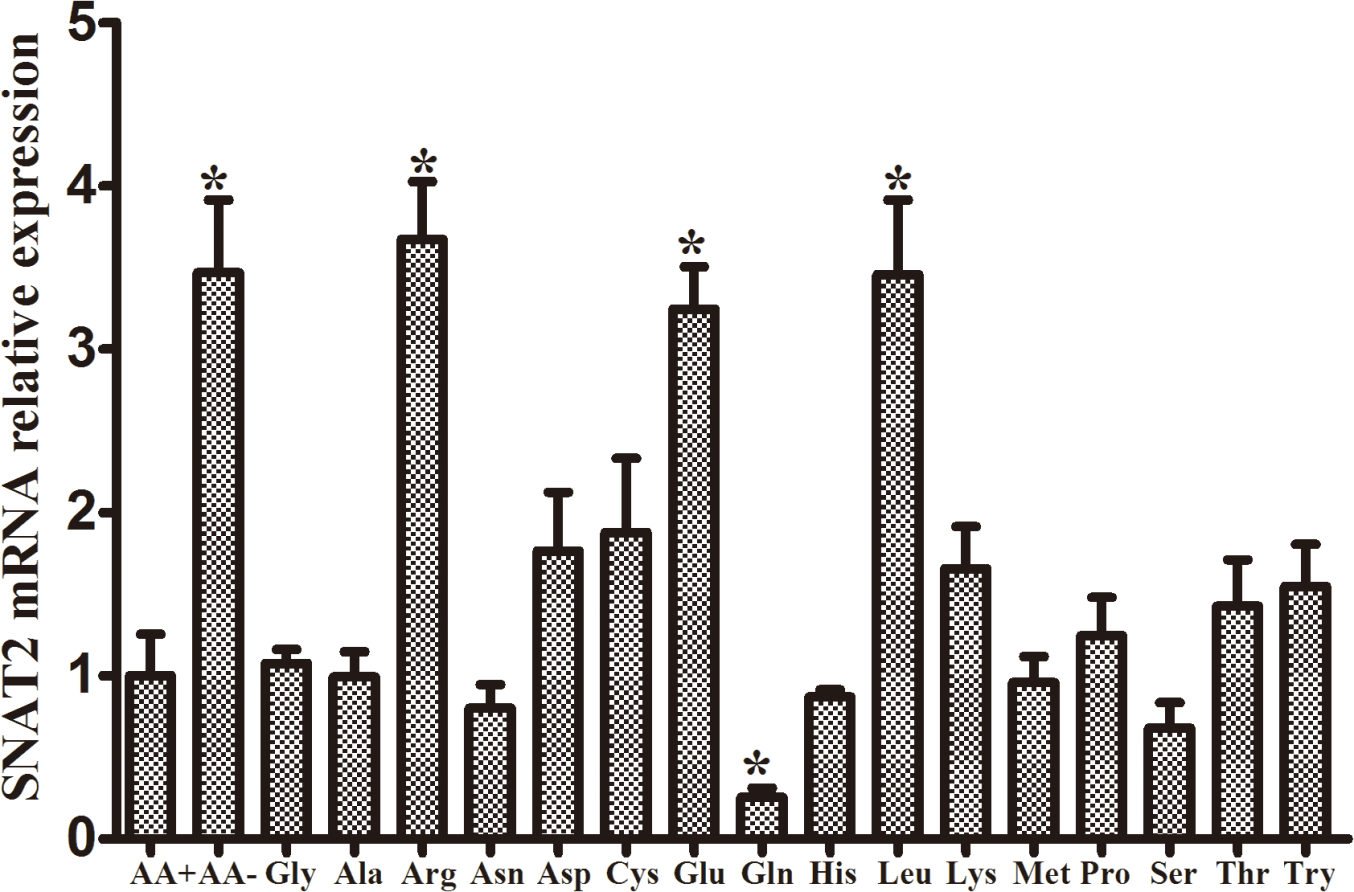
The relative SNAT2 mRNA expression in IPEC-1. IPEC-1 cells were incubated for 8 h in EBSS for amino acid-free (AA-) or in EBSS supplemented with amino acid mix (AA+) or individual amino acids based on the formulation of DMEM/F12 for cell stimulation. The relative SNAT2 mRNA expression was determined by real-time quantitative PCR using β-Actin as a reference gene. Data are expressed as means ± SEM, n = 4. * *P* < 0.05 versus amino acid- supplemented (AA+) cells.

As shown in Fig 7, amino acid starvation and supplementation with glutamate, arginine or leucine enhanced, while supplementation with glutamine reduced, SNAT2 mRNA levels in IPEC-1 (*P* < 0.05). SNAT2 influences the activity of transport system A for neutral amino acids such as glutamine, proline, and alanine [46], and its regulation is mediated particularly by extracellular amino acid concentrations. Amino acid deprivation has been shown to stimulate the functional expression of SNAT2 in rat L6 myotubes and HeLa cells [8], neocortical neurons, and trophoblast cells [47]. This is the first study to demonstrate that SNAT2 is subject to adaptive regulation following amino acid deprivation in enterocytes of piglets. However, system A substrates may suppress the increase in SNAT2 expression that occurs in AA-starved cells [8,47,48]. Glutamine has demonstrated to inhibit SNAT2 expression and the induction of ATF4 (activating transcription factor 4) and CCAAT/enhancer-binding protein which are known to bind to an amino acid response element within the SNAT2 promoter in neocortical neurons. Glutamine, a major nutrient utilized by the intestinal epithelium [4], also inhibited SNAT2 mRNA expression. In addition, the adaptive regulation of SNAT2 is not limited to the effects of system A substrates. Phenylalanine, tyrosine, and tryptophan also suppress system A [35], while glutamate, arginine and leucine enhance SNAT2 mRNA expression (Fig 7), which may be due to the function of SNAT2 as a nutrient receptor [8].

### Conclusions

*SLC38A2* from the small intestine was cloned and could be detected in the duodenum, jejunum and ileum of piglets. During the suckling period from days 1 to 21, the duodenal mucosa had the highest abundance of SNAT2 mRNA among the three segments of the small intestine of piglets. In the first week post-weaning, there was a significant decrease in the expression of SNAT2 mRNA in the duodenal and jejunal mucosa and in protein expression in the jejunal and ileal mucosa on day 1 after weaning. Amino acid starvation and supplementation wihth glutamate, arginine or leucine enhanced, while supplementation with glutamine reduced, SNAT2 mRNA expression. These results should be useful for further investigations of the mechanism of amino acid utilization and signal transduction mediated by SNAT2 in the intestine.
